# Early Challenges in the Implementation of Automated CranialRebuild Freeware for Generation of Patient-Specific Cranial Implant Using Additive Manufacturing: A Pilot Project in Review

**DOI:** 10.3390/biomimetics9070430

**Published:** 2024-07-16

**Authors:** Oleksandr Strelko, Manish Raj Aryal, Abigail Zack, Yara Alfawares, Roland Remenyi, Ian Kristopher Bayan, Yumi L. Briones, Yaroslav Holovenko, Maksym Maksymenko, Andrii Sirko, Sam Anand, Jonathan A. Forbes

**Affiliations:** 1 Stritch School of Medicine, Loyola University Chicago, Maywood, IL 601611, USA; ostrelko@luc.edu; 2 Center for Global Design and Manufacturing, Department of Mechanical Engineering, University of Cincinnati, Cincinnati, OH 45219, USA; 3 College of Medicine, University of Cincinnati, Cincinnati, OH 45219, USA; 4 Biomedical Research Unit, Clinical and Translational Research Institute, The Medical City, Pasig 1600, Philippines; 5 Department of Neurosurgery, Institute of The Neurological Sciences, The Medical City, Pasig 1600, Philippines; 6 3D Metal Tech LLC., 04114 Kyiv, Ukraine; 7 Center for Cerebral Neurosurgery, Mechnikov Dnipropetrovsk Regional Clinical Hospital, 49005 Dnipro, Ukraine; 8 Department of Neurosurgery, College of Medicine, University of Cincinnati, Cincinnati, OH 45219, USA

**Keywords:** cranioplasty, freeware, cranial implant, manufacturing, Traumatic Brain Injury, CranialRebuild

## Abstract

Traumatic Brain Injury (TBI) is a significant global health concern, particularly in low- and middle-income countries (LMICs) where access to medical resources is limited. Decompressive craniectomy (DHC) is a common procedure to alleviate elevated intracranial pressure (ICP) following TBI, but the cost of subsequent cranioplasty can be prohibitive, especially in resource-constrained settings. We describe challenges encountered during the beta-testing phase of CranialRebuild 1.0, an automated software program tasked with creating patient-specific cranial implants (PSCIs) from CT images. Two pilot clinical teams in the Philippines and Ukraine tested the software, providing feedback on its functionality and challenges encountered. The constructive feedback from the Philippine and Ukrainian teams highlighted challenges related to CT scan parameters, DICOM file arrays, software limitations, and the need for further software improvements. CranialRebuild 1.0 shows promise in addressing the need for affordable PSCIs in LMICs. Challenges and improvement suggestions identified throughout the beta-testing phase will shape the development of CranialRebuild 2.0, with the aim of enhancing its functionality and usability. Further research is needed to validate the software’s efficacy in a clinical setting and assess its cost-effectiveness.

## 1. Introduction

Traumatic Brain Injury (TBI) continues to be a significant public health burden across the world, with a global incidence of over 25 million cases annually. This burden is heavier in low- and middle-income countries (LMIC), particularly those in Central Asia and Eastern Europe, due to decreased access to sufficient medical resources that would otherwise prevent morbidity and mortality from treatable TBI [1]. Decompressive craniectomy (DHC) is often recommended to relieve elevated intracranial pressure (ICP) following TBI [2]. In addition to its importance in lowering TBI mortality rates, early cranioplasty has also been associated with improved functional outcomes in patients with severe TBI [3,4,5,6,7]. However, with an average cranioplasty cost ranging from USD 23,000 to USD 38,000 in more recent studies for customized cranial implants in the United States, this may be unaffordable in countries with limited resources and underdeveloped healthcare systems [8,9,10]. 

To overcome the cost barrier, various groups have utilized computer-assisted design and 3D printing to produce PSCIs at a lower price point [4,11,12,13,14]. However, in doing so, they have introduced a new barrier arising secondary to the complexity of the required software and the need for extensive training and time spent designing PSCIs. This knowledge barrier served as the motivation for the design of CranialRebuild, a fully automated software with a complete workflow starting from CT after decompressive hemicraniectomy to a 3D-printable STL file of a PSCI mold [15]. CranialRebuild 1.0 was tested in a cadaveric model and achieved hermetically precise PSCIs requiring moderate to minimal modification for optimal cosmesis at only 30% of the typical cost of PSCIs in the United States [14,16]. This success prompted further interest and pursuit of utilizing the CranialRebuild 1.0 application in LMICs. Here, we provide a narrative report describing the challenges encountered and optimizations implemented following the initiation of software beta-testing by medical teams at two distinct sites in the Philippines and Ukraine. 

## 2. Materials and Methods

Through discussions with the team of authors, pilot clinical teams with interest in testing the CranialRebuild 1.0 software were identified, namely, one team in the Philippines and one team in Ukraine. The CranialRebuild executable files were shared with each team, along with a simple instruction manual on its operation and a corresponding video to demonstrate its use [17]. With this information, each team was able to run CranialRebuild, and Digital Imaging and Communications (DICOM) files containing CT scans of post-decompressive hemicraniectomies obtained at their institutions as input for the program on their personal or work computers. Our team of medical professionals and engineers was available through email correspondence and virtual meetings to answer questions that arose throughout the process. Following this, the authors were able to elicit feedback from the teams regarding the functionality of the software and possible avenues of improvement. All CT scans were collected with appropriate institutional permissions obtained as required by the respective teams. 

**Figure 1:**
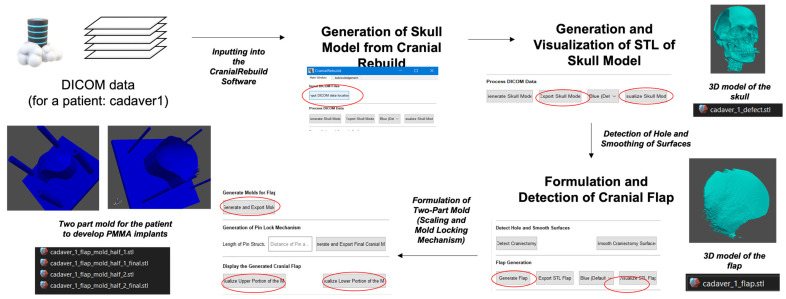
Workflow for the creation of a patient-specific cranial flap.

The functionality of the program has been described previously by Venugopal et al., but briefly, the program processes the input DICOM file by creating a virtual model of the skull, mirroring the intact side of the cranium onto the DHC side to identify the precise curvature of both outer and inner tables of the bone, and from this creates a virtual model of a PSCI to fill the cranial defect. A negative of the modeled PSCI is then generated to serve as a 3D-printable mold from which an actual implant may be created (Figure 1) [16]. CranialRebuild 1.0 is a freeware program available for download after an application requesting the use of the program is submitted to the Center for Global Design and Manufacturing at the University of Cincinnati through the following link: https://forms.office.com/Pages/ResponsePage.aspx?id=bC4i9cZf60iPA3PbGCA7Y_6oA5tqD_BFqrbI_odwhEFUODAwMERSTFFRWDVSV1pCUVVZVEFZSDY5Ni4u (US patent US20230414367A1, 28 December 2023). 

## 3. Results

The beta version of CranialRebuild 1.0 enables the creation of skull models, implants, and molds, offering three distinct applications for the software. The collaborators were encouraged to explore and investigate the software, assessing its capability as a self-sufficient, automated tool for generating cranial implants within a clinical environment. 

### 3.1. Feedback from the Philippine Team 

Our collaborators in the Philippines faced several challenges with the CranialRebuild 1.0 software, and by the time of this publication, they had not yet been able to generate STL files of implants or molds. However, in response to the challenges encountered thus far, the CranialRebuild team proposed a series of short-term and long-term solutions (Table 1), paving the way for the introduction of CranialRebuild 2.0. 

The first challenge encountered was in regard to the specific parameters of the CT scans used. CranialRebuild 1.0 was initially programmed to recognize a CT scan slice thickness ranging from 0.1 to 10 mm. Hence, the input of files with a slice thickness of 142.89 mm resulted in a program error. The Philippine team was advised to adjust the settings during CT scan acquisition and during the file export from the CT scanner software (Siemens Healthcare Inc., Makati, Philippines) to address this error in the short term. In the long term, the slice thickness should be specified as a requirement for using CranialRebuild in order to avoid compromising the precision of the designed implants or molds. 

Further challenges were encountered due to variations in the DICOM file construction. Specifically, the DICOM files in our cadaveric testing of the program stored bone and soft tissue data in one folder, which CranialRebuild extracted; in contrast, the DICOM files used by the Philippine team stored the bone and soft tissue data in two separate folders. Moreover, our initial DICOM files stored each CT scan slice in an array or table, and the arrays remained the same size throughout the DICOM file. In contrast, the Philippine team’s DICOM files used smaller arrays for slices where the cross-sectional image of the skull was smaller. This inconsistency in the array size was not well tolerated by CranialRebuild 1.0. In the short term, the Philippine team was instructed to export the bone data into a single DICOM folder using the export function of the CT software; nevertheless, the challenge with the variation in array size continued to hinder progress. When the Philippine team investigated the files within the re-exported DICOM folder, they noted that each exported folder contained one file with a different file size, usually the first file in each series. After the removal of this file and uploading the DICOM folder to CranialRebuild, the Philippine team could successfully generate and export skull models. Nonetheless, they encountered further errors at the subsequent step of implant creation. Our eventual goal is to release an update of CranialRebuild 2.0 to automatically select the largest folder in a DICOM file, which has been found to typically contain bone data, allowing it to process DICOM files with bone and soft tissue data combined or separated. It will also incorporate an advanced pre-check feature, allowing users to select an array size that is applied to all arrays in their DICOM file. 

**Figure 2:**
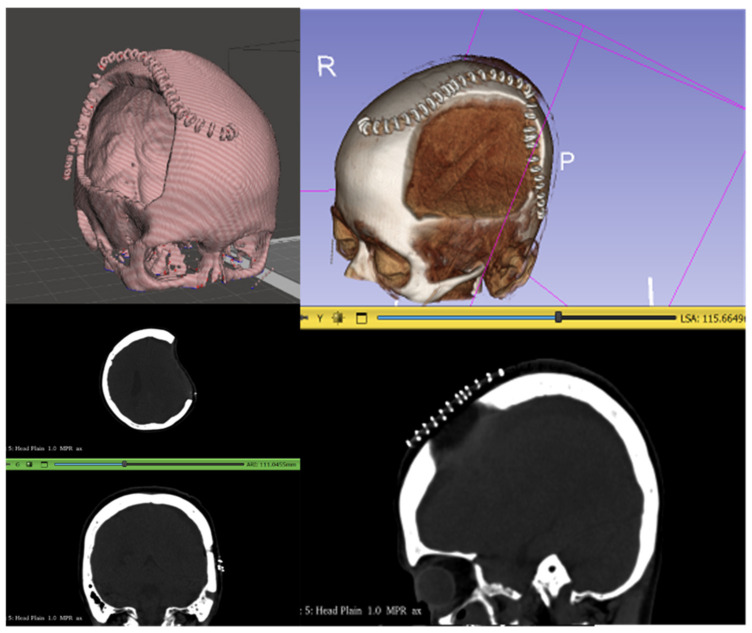
Staples overlying the DHC site were detected by CranialRebuild as bone, hindering its ability to correctly identify the midsagittal plane of the skull for further processing.

In addition to the abovementioned challenges, the Philippine team faced a problem related to surgical staples used for incision closure after DHC. CranialRebuild 1.0 detected these as bone fragments due to their similar material density on the CT scan; the software used the staple structures to determine the midsagittal plane through which it would reflect the intact cranium onto the DHC side, resulting in an error (Figure 2). This will ultimately be addressed by recommending that the CT scan be acquired after staple removal, which is typically 2 weeks after DHC, but before cranioplasty. 

### 3.2. Feedback from the Ukrainian Team 

The Ukrainian collaborators, in parallel with the efforts of the Philippine team, rigorously evaluated CranialRebuild 1.0, contributing valuable insights to refine its functionality. They were able to complete all of the steps of CranialRebuild to produce 3D-printed molds; however, they were also interested in using CAD software to apply minor modifications to the CranialRebuild-designed implants, as their team included engineers with the required training to do so. The one challenge experienced by this team during testing was the issue with inconsistent array size, just as experienced by the Philippines team. In this case, it was overcome by reacquiring the DICOM files with a consistent array size. This challenge aside, the team’s suggestions for software improvement are outlined below (Table 2). A prioritization scale was developed by our team, ranging from 1 to 5 (1 = highest priority, 2 = high priority, 3 = medium priority, 4 = low priority, 5 = lowest priority), to prioritize suggestions that would improve the functionality for all users over suggestions tailored to this team’s specific interest for further CAD processing of the implants. 

One suggestion was to develop a functionality where CranialRebuild 2.0 would allow the user to visualize the designed implant as it would fit into the DHC site in virtual 3D space before 3D printing. This request was assigned a priority of 4, indicating that it would require a low–medium level of effort, as determined by our engineers, but would be a highly valuable feature. 

Another suggestion was to replace the original CranialRebuild scaling feature, which scaled the designed implant down in size by 3% (empirically determined value through cadaveric testing) before printing. The Ukrainian team suggested this be replaced with a feature where the software would align one edge of the designed implant, either the anterior or posterior, with the DHC edge, allowing their engineers to subsequently trim any remaining overlap with the bone from the implant. This request earned a priority level of 2, as our engineers determined the change would require considerable effort and the result would be tailored to the Ukrainian team’s workflow and their access to engineers trained in CAD. However, our team recognizes that an option for scaling or trimming might be valuable in the future. 

**Figure 3:**
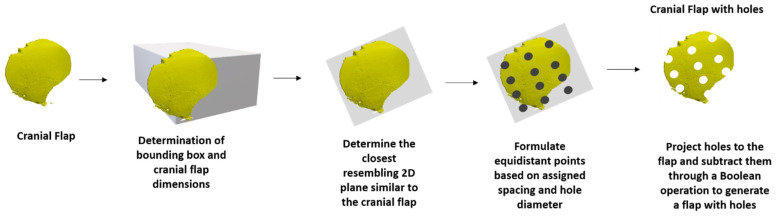
Generation methodology for developing cranial flap model with uniform holes.

Finally, the Ukrainian team proposed an added detail where CranialRebuild would include a grid of small holes in the design of the implants to serve as outlets, which would prevent fluid accumulation and increased intracranial pressure. CranialRebuild 2.0 was updated to overlay a grid with 3 mm holes spaced 15–25 mm apart onto the designed implants (Figure 3), as is standard for implants currently in clinical use (Stryker, Anatomics, Portage, MI, USA). The Ukrainian team was specifically interested in this as it would allow for the direct 3D printing of CranialRebuild implants out of polyetheretherketone (PEEK) or titanium alloy rather than printing a mold and using polymethyl methacrylate (PMMA) to produce the implant, as was carried out in our cadaveric testing. 

## 4. Discussion

To continually improve CranialRebuild’s functionality, we collaborated with teams in the Philippines and Ukraine that were interested in testing our software, as outlined in this narrative review. 

The Philippine team faced some challenges, which have partly been resolved but have highlighted areas of improvement to consider when engineering CranialRebuild 2.0. The major theme of these challenges was the data format of the CT scans and DICOM files. The slice thickness of the CT scans used by this team was significantly larger than that initially tested by our team and accepted by CranialRebuild. Slice thickness is typically determined by the thickness of the compartments of the CT scanner detector, which is a fixed size, and, subsequently, by the thickness selected by the technician during multiplanar reconstruction, which can be highly variable. Despite this variation, CT slice thickness is commonly kept below 10 mm to increase the image resolution [15,18]. This limit is consistent with our recommendation to maintain the precision of the final product. The remaining challenges that faced the Philippine team were the separation of bone and soft tissue data and the variation in the array size, which could be a common problem, as DICOM files do not have a fixed array size [19]. The issue of data separation will be addressed by a pre-check feature in CranialRebuild 2.0, which will be able to process both combined and separated data. The problem of varying array size will be addressed by allowing future users to select an appropriate array size for their data, which may be applied to all arrays in a DICOM file to ensure uniformity. 

While the Ukrainian team also faced the challenge of varying array size, they were later able to advance through the steps of CranialRebuild and successfully generate the implant models. From their experience, they made insightful suggestions to consider when engineering CranialRebuild 2.0, including fitted implant and skull virtual modeling, an alternative option for post-design resizing of the implants, and the introduction of outlet holes into the implants. Notably, the Ukrainian team beautifully highlighted the broader functionality of our freeware with its possibilities for use in designing implants or molds, which could either be immediately printed or alternatively used as the basis for further CAD modification, if resources allow. This post-design minimal modification can be carried out manually after implant printing or molding, as described in our previous publication, not undermining the ability of teams without CAD knowledge to use CranialRebuild [17].

Overall, the global demand for affordable 3D-printed PSCIs is evident in the abundance of advancement made in the field by various contributors [4,12,13,14]. For example, a Canadian team testing the OR cost effectiveness of PMMA implants found the cost to range from CAD 342 to 620, which included the cost of 3D printing and the PMMA material for the implant. This cost is significantly less than the typical CAD 15,000 for a commercially available PSCI, demonstrating the measurable price difference when 3D implants are utilized [20]. If using the PMMA price listed by Ashraf et al., CranialRebuild PSCIs may also be produced at as low a price as CAD 40 per implant after the initial investment in a 3D printer [12]. However, it bears mentioning that estimations for the total cost of a PSCI created with CranialRebuild have all been performed in USD or CAD, so further research on the exact cost of the implants in LMICs where this could be of great use is necessary. Furthermore, CranialRebuild remains the only freeware to fully automate the process of data manipulation and implant or mold design, eliminating the need for extensive time, training, and clinical expertise otherwise required to manually generate molds using other available freeware [21]. In addition, the versatility of CranialRebuild allows its application to generate implants from a variety of materials, including PMMA, PEEK, and titanium, allowing for broader clinical coverage [22]. Finally, creating a PSCI from PMMA prior to surgery allows additional time to modify and shape the implant more precisely, decreasing operative time and preventing complications resulting from improper implant shape and size [12].

The utility of this software is marked in LMICs due to the heavier burden of TBI and decreased access to the necessary medical care, especially when this becomes exacerbated by war, as is currently the case in Ukraine [1]. This software’s continued improvement and dissemination is anticipated to greatly improve access to cosmetically and medically sound implants in countries with great need.

### Limitations

Although the software has been tested in cadaveric specimens and feedback has been received from collaborators in the Philippines and Ukraine, the development of CranialRebuild has not yet advanced to clinical testing in patients. Widespread adoption should only occur once a larger body of evidence exists to support the program’s efficacy. Further improvements to the freeware should focus on developing a tolerance for variation in the data formatting and minimizing the need for post-design modifications. In addition, a detailed cost analysis should be performed in countries where the software may be of most use when implemented, as the cost is currently based on figures from the United States. 

## 5. Patents

Venugopal V, McConaha M, Ghalsasi O, Xu A, Anand S, Forbes J, Cheng J, Aryal MR, inventors; University of Cincinnati, assignee. Method of designing patient-specific cranioplasty implants. United States patent application US 18/212,873, 28 December 2023.

## Figures and Tables

**Table 1:** Overview of challenges encountered by the Philippine team during testing of CranialRebuild 1.0 and corresponding short-term and long-term solutions to be incorporated into the design of CranialRebuild 2.0.

Challenges	Brief Description	Short-Term Solution	Long-Term Solution
Variation in CT scan slice thickness.	The Philippine team used CT scans with a slice thickness of 142.89 mm, while CranialRebuild 1.0 was designed to accept a slice thickness ranging from 0.1 to 10 mm.	The Philippine team was advised to adjust for this requirement in slice thickness during CT scan acquisition.	The slice thickness requirement of 0.1–1 mm will be specified for the future use of CranialRebuild to not compromise the precision of the product.
Bone and soft tissue data storage in separate folders.	While the DICOM files used in our cadaveric testing stored bone and soft tissue data in a combined folder, the data used by the Philippine team were separated into two distinct folders.	The Philippine team had to re-export the bone CT data into a single DICOM folder, which could be input to CranialRebuild.	CranialRebuild will be updated to automatically extract the largest folder, which has been found to typically store the bone data regardless of whether the soft tissue data are also stored in that folder.
Inconsistency in array size in a single DICOM file.	The DICOM files used by the Philippine team matched the array size to the physical size of the skull in cross-section through each CT scan slice, causing arrays of different sizes.	This continues to pose a barrier to progress in testing at the time of publication of this paper.	CranialRebuild 2.0 will be engineered to allow the user to select the size of their largest array, and subsequently resize all other arrays to ensure size consistency before further processing.
Misidentification of surgical staples as bone.	Some CT scans included surgical staples overlying the DHC and were detected by CranialRebuild as bone, which impacted the software’s determination of the midsagittal plane.	The recommendation was made to acquire CT scans after staple removal (typically 2 weeks after DHC) but before cranioplasty (2–3 months after DHC).	This specific timing of the CT scan should be recommended in the future use of CranialRebuild 2.0.

**Table 2:** Overview of challenges and suggestions made by the Ukrainian team during testing of CranialRebuild 1.0 with our team’s comments and assigned priority level (1 = highest priority, 2 = high priority, 3 = medium priority, 4 = low priority, 5 = lowest priority).

Suggestion/Challenge	CranialRebuild Team Comments	Priority
Inconsistency in array size in a single DICOM file.	Effort required to address: Medium. Ability to improve software: Highly valuable as the Philippine team encountered the same issue.	5
Add ability to visualize implant fitted onto DHC site in virtual modeling before 3D printing.	Effort required to address: Medium. Ability to improve software: Highly valuable for 3D model visualization.	4
Add option to align the implant with one edge of the DHC site and trim any overlap with the bone.	Effort required to address: High. Ability to improve software: Valuable in the long run, as some neurosurgeons might prefer this to our original scaling method.	2
Add the detail of including a grid of outlet holes onto the designed implant.	Effort required to address: High. Ability to improve software: Valuable as it is more in line with current clinically used implants and would allow future users the option of directly printing implants rather than molds.	4

## Data Availability

The raw data supporting the conclusions of this article will be made available by the authors on request.
